# Supplementary material for: Optimizing test and treat options for vivax malaria: An options assessment toolkit (OAT) for Asia Pacific national malaria control programs
Source: PLOS Glob Public Health. 2024 May 22;4(5):e0002970. doi: 10.1371/journal.pgph.0002970 (PMC11111040; doi:10.1371/journal.pgph.0002970)
Supplement: S1 Table — (PDF) [file pgph.0002970.s001.pdf]

S1 Table: Demographic characteristics of responding experts for BAT modified e-Delphi

Characteristics		Round one		Round two	
		**Number (n=21)**	**Percentage (%)**	**Number (n=20)**	**Percentage (%)**
Region	South East Asia	11	55	12	60
	Western Pacific	4	20	3	15
	Global	3	15	3	15
	Eastern Mediterranean	2	10	2	10
Gender	Female	13	62	12	60
	Male	8	38	8	40
Affiliation	Academic/Research institution	19	90	18	90
	Government Agency	1	5	1	5
	NGO	1	5	1	5
	Other	1	5	1	5
Expertise	Treatment of vivax malaria	16	76	15	75
	Malaria Epidemiology	16	76	15	75
	Diagnostics and Surveillance	14	67	13	65
	Pathology and Pathogenesis of vivax malaria	9	43	9	45
	Health Policy	4	19	4	20
	Entomology and Vector control	4	19	3	15
	Other	3	14	3	15
	Behavioral Science	1	5	1	5
