# Supplementary material for: Optimizing test and treat options for vivax malaria: An options assessment toolkit (OAT) for Asia Pacific national malaria control programs
Source: PLOS Glob Public Health. 2024 May 22;4(5):e0002970. doi: 10.1371/journal.pgph.0002970 (PMC11111040; doi:10.1371/journal.pgph.0002970)
Supplement: S2 Table — (PDF) [file pgph.0002970.s002.pdf]

S2 Table: Comparison of originally intended composition of toolkit and final composition

S. No	Tools	Status	Rationale
1	**Baseline assessment template (BAT***)* *previously named as Readiness assessment template.*	**Included**	Assess readiness of malaria program for vivax elimination.
2	**Scenarios** representative of Asia Pacific region	**Included**	Scenario representative of the region for epidemiological, health system and political economic context.
3	**Scenario based test and treat options**	**Included**	Optimal test and radical cure treatment options of vivax, based on the scenarios
4	**Step-by-step guidance** on how to use the OAT toolkit (based on documentation of the process and engagement with NMPs)	**Included**	Assists NMPs to use the toolkits
5	**Evidence briefs** on efficacy and effectiveness of current radical cure drugs and latest information on high sensitivity rapid diagnostic tests (HS-RDTs), G6PD screening tests, and radical cure options near end of pipeline	Abandoned	Evidence is continuously evolving and new evidence needs to be added continuously to revise OAT.
6	NMP **weighting tool** for different variables	Abandoned	Too complex, limited data available
7	**Approaches** for optimized radical cure tools	**Included**	Outlines the policy change process and considerations for policy change and implementation.
8	Policy options evaluation matrix	Abandoned	Too complex and not user friendly
9	Policy uncertainties and potential mitigation actions template	Abandoned	Too complex, data may not be available
10	**Decision tree**	Abandoned	NMPs felt it would not be useful
