# Supplementary material for: Optimizing test and treat options for vivax malaria: An options assessment toolkit (OAT) for Asia Pacific national malaria control programs
Source: PLOS Glob Public Health. 2024 May 22;4(5):e0002970. doi: 10.1371/journal.pgph.0002970 (PMC11111040; doi:10.1371/journal.pgph.0002970)
Supplement: S3 Table — (PDF) [file pgph.0002970.s003.pdf]

S3 Table: The 25 factors initially included in the BAT and the reasons for their inclusion or exclusion

S. No	Factors	Status	Rationale
**Epidemiological domain**			
1.	Vivax malaria caseload	Added after NMP feedback	Representation of phases of malaria program
2.	Geographic variations in vivax cases within the country	Excluded after NMP feedback	Influences implementation decision and approaches but less relevant for policy decisions.
3.	Efficacy and Effectiveness of current radical cure treatment regimen	Retained after NMP feedback	Given context may influence policy and implementation decisions
4.	Vulnerable populations at risk	Excluded after NMP feedback	Policy is prepared to address wider population
5.	Vivax relapse periodicity (predicted) (*additional factor**)	Excluded after NMP feedback	Limited information with NMPs
6.	Chloroquine resistance (*additional factor**)	Excluded after NMP feedback	Limited information with NMPs
7.	G6PD deficiency prevalence	Retained after NMP feedback	Given context may influence policy on radical cure.
**Implementation domain**			
8.	Access to radical cure treatment regimen	Retained after NMP feedback	Assess the strength of health system
9.	Coverage of current radical cure regimen	Excluded after NMP feedback	Does not influence vivax radical cure policy
10.	Healthcare worker adherence to guidelines	Retained after NMP feedback	Assess the strength of health system
11.	Patient adherence	Retained after NMP feedback	
12.	Pharmacovigilance	Retained after NMP feedback	
13.	Logistics and supply chain	Excluded after NMP feedback	Does not influence vivax radical cure policy
14.	Human resources	Retained after NMP feedback	
15.	Quality of training and supervision to healthcare workers	Excluded after NMP feedback	Does not influence vivax radical cure policy

S. No	Factors	Status	Rationale
**Enabling - Political and economic domain**			
16.	Antimalarial policy change processes	Excluded after NMP feedback	Important to map out but not through the baseline assessment
17.	Political will for vivax elimination	**Retained after NMP feedback**	Commitment of NMPs for elimination target
18.	Acceptance/ interest/appetite for alternative solutions with different risk/benefits to current regimes	**Retained after NMP feedback**	Influences vivax radical cure policy and included as factor "risk aversion"
19.	Ease of policy implementation	Excluded after NMP feedback	Important to map out but not through the baseline assessment
20.	Administrative feasibility	Excluded after NMP feedback	Does not explicitly influence vivax radical cure policy
21.	Economic burden of vivax	Excluded after NMP feedback	Important to map out but not through the baseline assessment
22.	Cost-effectiveness analysis of radical cure tools	Excluded after NMP feedback	Relevant for later tools developed
23.	Public spending for malaria	Retained after NMP feedback	Combined as a single factor of 'Budget"
24.	External donor funding	Retained after NMP feedback	
25.	Income inequality	Excluded after NMP feedback	Does not explicitly influence vivax radical cure policy

*additional factors were suggested in the development phase by the initial core team, based on literature reviews and discussions.
