# Supplementary material for: Optimizing test and treat options for vivax malaria: An options assessment toolkit (OAT) for Asia Pacific national malaria control programs
Source: PLOS Glob Public Health. 2024 May 22;4(5):e0002970. doi: 10.1371/journal.pgph.0002970 (PMC11111040; doi:10.1371/journal.pgph.0002970)
Supplement: S4 Table — (PDF) [file pgph.0002970.s004.pdf]

S4 Table: Ranking of factors included in the BAT by the NMP participants

**Country**		**Solomon Islands**	**Afghanistan**	**Vietnam**
	**Specific factors**			
1. How do you rate the importance of this factor/question for assessment of your readiness for vivax elimination?	phase of malaria program	High	High	High
2. How do you rate the importance of this factor/question for your assessment of readiness for vivax elimination?	vivax case load	High	High	High
3. How do you rate the importance of this factor/question for assessment of your readiness for vivax elimination?	G6PD prevalence	High	Low	High
4. How do you rate the importance of this factor/question for assessment of your readiness for vivax elimination?	G6PD def. heterogeneity	High	High	High
5. How do you rate the importance of this factor/question for assessment of your readiness for vivax elimination?	Blood stage treatment	High	Moderate	High
6. How do you rate the importance of this factor/question for assessment of your readiness for vivax elimination?	liver stage treatment	High	High	High
7. How do you rate the importance of this factor/question for assessment of your readiness for vivax elimination?	Antirelapse efficacy	High	High	High
8.1 How do you rate the importance of this factor/question for assessment of your readiness for vivax elimination?	functioning of referral system - referral initiation	Moderate	Moderate	High
8.2 How do you rate the importance of this factor/question for assessment of your readiness for vivax elimination?	functioning of referral system - referral completion	Moderate	moderate	High
9.1 How do you rate the importance of this factor/question for assessment of your readiness for vivax elimination?	Patient adherence - proportion	High	High	High
9.2 How do you rate the importance of this factor/question for assessment of your readiness for vivax elimination?	patient adherence - supervised	Moderate	High	High

**Country**		**Solomon Islands**	**Afghanistan**	**Vietnam**
10. How do you rate the importance of this factor/question for assessment of your readiness for vivax elimination?	human resource-health workers available	High	High	High
11. How do you rate the importance of this factor/question for assessment of your readiness for vivax elimination?	Pharmacovigilance	High	High	Moderate
12. How do you rate the importance of this factor/question for assessment of your readiness for vivax elimination?	budget	Moderate	High	Moderate
13. How do you rate the importance of this factor/question for assessment of your readiness for vivax elimination?	political will	Moderate	Moderate	High
14. How do you rate the importance of this factor/question for assessment of your readiness for vivax elimination?	Risk aversion	High	High	High
