# Supplementary material for: Optimizing test and treat options for vivax malaria: An options assessment toolkit (OAT) for Asia Pacific national malaria control programs
Source: PLOS Glob Public Health. 2024 May 22;4(5):e0002970. doi: 10.1371/journal.pgph.0002970 (PMC11111040; doi:10.1371/journal.pgph.0002970)
Supplement: S5 Table — (PDF) [file pgph.0002970.s005.pdf]

S5 Table: Results from round one of the first modified e-Delphi to validate the factors included in the BAT

Factor	Number of respondents who think the factor is important for readiness assessment and/or decision making on test and treat combinations	% Agreement	Threshold agreement achieved
**1) Epidemiological Domain**			
i) Phase of malaria program	19	90	Yes
ii) Vivax caseload	20	95	Yes
iii) G6PD deficiency prevalence	21	100	Yes
iv) G6PD deficiency heterogeneity	21	100	Yes
v) Blood stage treatment	18	86	Yes
vi) Liver stage treatment	19	90	Yes
vii) Antirelapse efficacy	20	95	Yes
**2) Implementation Domain**			
i) a. Referral system (referral initiation rate)	21	100	Yes
b. Referral system (referral completion rate)	20	95	Yes
ii) a. Human resource (available in community)	21	100	Yes
b. Human resource (HW compliance rate)	21	100	Yes
iii) a. Patient adherence (adherence rate)	21	100	Yes
b. Patient adherence (supervised treatment)	21	100	Yes
iv) Pharmacovigilance	20	95	Yes
**3) Enabling Domain**			
i) Budget	18	86	Yes
ii) Political will	13	**62**	**No**
iii) Risk aversion	19	90	Yes
