# Supplementary material for: Optimizing test and treat options for vivax malaria: An options assessment toolkit (OAT) for Asia Pacific national malaria control programs
Source: PLOS Glob Public Health. 2024 May 22;4(5):e0002970. doi: 10.1371/journal.pgph.0002970 (PMC11111040; doi:10.1371/journal.pgph.0002970)
Supplement: S6 Table — (PDF) [file pgph.0002970.s006.pdf]

S6 Table: Response on the factors in round two of the first modified e-Delphi

Factor	Number of respondents who think the factor is important for readiness assessment and/or decision making on test and treat combinations	Total respondents who answered yes/no	% Agreement	Threshold agreement achieved
**I) Epidemiological Factors**				
i) Severity of G6PD deficiency*	15	19	79	Yes
ii) Safety of radical cure regimen*	16	19	84	Yes
**II) Implementation Factors**				
i) Feasibility of evidence use*	20**	20	100	Yes
**III) Enabling Factors**				
i) Political will	14	18	78	Yes

**Additional factor suggested in round one included in round two to reach agreement*

*** if response= Yes: it ranges from somewhat to a lot (somewhat: 6/20,30%; moderate: 7/20, 35%; a lot: 7/20, 35%)*
