# Supplementary material for: Optimizing test and treat options for vivax malaria: An options assessment toolkit (OAT) for Asia Pacific national malaria control programs
Source: PLOS Glob Public Health. 2024 May 22;4(5):e0002970. doi: 10.1371/journal.pgph.0002970 (PMC11111040; doi:10.1371/journal.pgph.0002970)
Supplement: S7 Table — (PDF) [file pgph.0002970.s007.pdf]

S7 Table: Additional factors suggested by experts

Additional factors suggested by experts in round 1	Status	Rationale	Status in round 2 Delphi
Vivax case heterogeneity	Not taken forward to round two after internal discussion	Influence on implementation decision and approaches but less on policy related to vivax radical cure	
Severity of G6PD deficiency	Retained for round 2	Important for radical cure treatment policy, challenges lie with data availability	Experts agreed on its importance
Safety of radical cure	Retained for round 2	Affects NMPs decision making process on vivax radical cure	Experts agreed on its importance
Feasibility of evidence use	Retained for round 2	Important on considering policy changes and its implementation	Experts agreed on its importance
Variant type of enzyme CYP2D6 gene	Not taken forward to round two after internal discussion	Limited data with NMPs	
Insecticide resistance	Not taken forward to round two after internal discussion	Important for overall malaria control but less relevant for treatment policy change	
Quality of malaria commodities	Not taken forward to round two after internal discussion	Important for overall malaria control but less relevant for treatment policy change	
Existence of community outreach.	Not taken forward to round two after internal discussion	Important for overall malaria control, addressed by the factors in the implementation factors	
