# Supplementary material for: Optimizing test and treat options for vivax malaria: An options assessment toolkit (OAT) for Asia Pacific national malaria control programs
Source: PLOS Glob Public Health. 2024 May 22;4(5):e0002970. doi: 10.1371/journal.pgph.0002970 (PMC11111040; doi:10.1371/journal.pgph.0002970)
Supplement: S8 Table — (PDF) [file pgph.0002970.s008.pdf]

S8 Table: Responses on the questions in each factor/ additional factor BAT (Round two of the first modified e-Delphi)

Factor	Question	Total respondents who answered yes/no	Number of respondents who agree that the question/categorization adequately captures the factor	% Agreement	Threshold agreement achieved
Severity of G6PD deficiency	What level of severity of G6PD deficiency is the most common in your country?	13	8	**61%**	**No**
Political will	13.a Do you think the question "Who was the chief guest in the last World Malaria Day event in your country?" is adequate for capturing this factor?	14	5	**36%**	**No**
			Central tendency	Range	
Antirelapse efficacy	adequate antirelapse efficacy (defined as risk of recurrence and not risk/ probability of recurrence free) at six months for decision-making for any given radical cure regimen	20	Mean: 85.5%	50-100%	Yes
Safety of radical cure regimen (Upper limit of SAE) *	Upper limit of severe hemolytic events requiring transfusion related to 8-aminoquinolines, adequate to consider it safe enough?	13	<1/100,000 = 6/13 (46%)<br><1/10,000 = 5/13 (38%)<br><1/1,000 = 2/13 (15%)	<1/100,000 – <1/1,000	NA

*this factor was initially considered important for BAT related to radical cure treatment but later it was removed citing the unavailability of data.
