# Supplementary material for: Optimizing test and treat options for vivax malaria: An options assessment toolkit (OAT) for Asia Pacific national malaria control programs
Source: PLOS Glob Public Health. 2024 May 22;4(5):e0002970. doi: 10.1371/journal.pgph.0002970 (PMC11111040; doi:10.1371/journal.pgph.0002970)
Supplement: S9 Table — (PDF) [file pgph.0002970.s009.pdf]

S9 Table: Detailed responses on the questions and categorizations in each factor BAT (Round one of the first modified e-Delphi)

Factor	Question	Categorization	Total respondents who answered yes/no	Number of respondents who agree that the question/categorization adequately captures the factor	% Agreement	Threshold agreement achieved
Phase of malaria program	1.a What is the phase of malaria program in your country?" is adequate for capturing this factor?		16	14	87%	Yes
		1.b We have classified Phase of malaria program into four categories, based on WHO, 2008, as:<br>a) Control (slide or RDT positivity rate ≥5),<br>b) Pre-elimination (slide or RDT positivity rate <5%),<br>c) Elimination (<1 case/1000 population at risk per year),<br>d) Prevention of reintroduction (3 years of 0 locally acquired cases)	13	10	77%	Yes
Vivax caseload	2.a Do you think the question "What is the number of annual reported cases of vivax in your country?" is adequate for capturing this factor?		18	14	78%	Yes.
		2.b We have classified Vivax caseload into seven categories, adapted from Battle & Baird, 2021, as:<br>a) >100000,	13	10	77%	Yes

Factor	Question	Categorization	Total respondents who answered yes/no	Number of respondents who agree that the question/categorization adequately captures the factor	% Agreement	Threshold agreement achieved
		b) 10001-100000,<br>c) 1001-10000,<br>d) 101-1000,<br>e) 1-100,<br>f) 0<br>g) Unknown				
G6PD deficiency prevalence	3.a Do you think the question "What is the level of G6PD deficiency (defined as less than 30% G6PD activity) in your country?" is adequate for capturing this factor?		17	14	82%	Yes
		3.a Do you think the question "What is the level of G6PD deficiency (defined as less than 30% G6PD activity) in your country?" is adequate for capturing this factor?	14	13	93%	Yes
G6PD deficiency heterogeneity	4.a Do you think the question "How would you describe the spatial heterogeneity of G6PD deficiency in your		18	14	78%	Yes

Factor	Question	Categorization	Total respondents who answered yes/no	Number of respondents who agree that the question/categorization adequately captures the factor	% Agreement	Threshold agreement achieved
	country?" is adequate for capturing this factor?					
		4.b We have classified G6PD deficiency heterogeneity into three categories as:<br>a) Heterogeneous,<br>b) Non-heterogeneous,<br>c) Don't know	12	10	83%	Yes
Blood stage treatment	5.a Do you think the question "What is the blood stage treatment used for uncomplicated vivax in your country?" is adequate for capturing this factor?		18	17	94%	Yes
		5.b We have classified Blood stage treatment into two categories as:<br>a) Chloroquine, and<br>b) Artemisinin Combination Therapy	15	15	100%	Yes
Liver stage treatment	6.a Do you think the question "What are the current radical cure regimen/s (one or more options) for uncomplicated		19	18	95%	Yes

Factor	Question	Categorization	Total respondents who answered yes/no	Number of respondents who agree that the question/categorization adequately captures the factor	% Agreement	Threshold agreement achieved
	vivax malaria recommended by the national treatment guidelines in your country?" is adequate for capturing this factor?					
		6.b We have classified the current recommended Liver stage treatment into six categories as:<br>a) PQ14days (0.25mg/kg/day for a total 3.5mg/kg),<br>b) PQ14days (0.5mg/kg/day for a total 7mg/kg),<br>c) PQ8weekly (0.75mg/kg/week for a total 6mg/kg),<br>d) PQ7days (0.5mg/kg/day for a total 3.5mg/kg)<br>e) None,<br>f) Others (specify)	16	14	87%	Yes
Antirelapse efficacy	7.a Do you think the question "Antirelapse efficacy data is available for which radical cure drug regimen/s in your country or similar settings?" is adequate for capturing this factor?		18	15	83%	Yes

Factor	Question	Categorization	Total respondents who answered yes/no	Number of respondents who agree that the question/categorization adequately captures the factor	% Agreement	Threshold agreement achieved
		7.d What should be an appropriate threshold (%) for adequate antirelapse efficacy (defined as risk of recurrence and not as risk/probability of recurrence free) at six months for decision-making for any given radical cure regimen?	17	Mean: 52.8<br>Range: 5-95		No*
Referral system						
Functioning of referral system (a. What is the estimated proportion of vivax patients referred from initial point of malaria diagnosis to higher centers?)	8.1a Do you think the question "What is the estimated proportion of vivax patients referred from initial point of malaria diagnosis to higher centers?" is adequate for capturing this factor?		19	18	95%	Yes
		8.1b We have classified Referral initiation rate into five categories, adapted from Measure Evaluation, 2013, as: a) <10%, b) >10-50%, c) >50-80%, d) >80-100%, and e) Don't know	15	15	100<br>%	Yes
Functioning of referral system (b.	8.2a Do you think the question "What is the		17	15	88%	Yes

Factor	Question	Categorization	Total respondents who answered yes/no	Number of respondents who agree that the question/categorization adequately captures the factor	% Agreement	Threshold agreement achieved
What is the estimated proportion of referred vivax patients that complete referral at receiving health facility?)	estimated proportion of referred vivax patients that complete referral at receiving health facility?" is adequate for capturing this factor?					
		8.2b We have classified Referral completion rate into five categories, adapted from Measure Evaluation, 2013, as: a) <10%, b) >10-50%, c) >50-80%, d) >80-100%, and e) Don't know	13	13	100%	Yes
Human resource						
Human resource (What kind of	9.1a Do you think the question "What kind of					

Factor	Question	Categorization	Total respondents who answered yes/no	Number of respondents who agree that the question/categorization adequately captures the factor	% Agreement	Threshold agreement achieved
health workers are available at the community level for malaria case management?)	health workers are available at the community level for malaria case management?" is adequate for capturing this factor?		20	16	80%	Yes
		9.1b We have classified Human resource into five categories as a) HW not available at community level;<br><br>b) HW available but cannot test, treat, or track;<br><br>c) HW available and can test and track but cannot treat;<br><br>d) HW available and can test, treat, and track for patient adherence<br><br>e) Don't know	15	12	80%	Yes
Human Resource (What do you think is the estimated proportion of health workers at different levels of the health system who adhere to current or new	9.2a Do you think the question "What do you think is the estimated proportion of health workers at different levels of the health system who adhere to current or new treatment protocols?" is adequate for capturing this factor?		18	14	78%	Yes

Factor	Question	Categorization	Total respondents who answered yes/no	Number of respondents who agree that the question/categorization adequately captures the factor	% Agreement	Threshold agreement achieved
treatment protocols?)		9.2b We have classified the estimated Health Worker compliance rate into four categories as:<br>a) <50%,<br>b) 50-80%,<br>c) >80%, and<br>d) Don't know	14	14	100%	Yes
Patient adherence						
Patient adherence (What do you think is the estimated proportion of patients who adhere to the full treatment regimen of the current recommended radical cure drugs?)	10.1a Do you think the question "What do you think is the estimated proportion of patients who adhere to the full treatment regimen of the current recommended radical cure drugs?" is adequate for capturing this factor?		21	20	95%	Yes
		10.1b We have classified the estimated patient adherence rate into four categories, adapted from Kim et al, 2018 and Burnier, 2019, as:<br>a) <50%,	17	17	100%	Yes

Factor	Question	Categorization	Total respondents who answered yes/no	Number of respondents who agree that the question/categorization adequately captures the factor	% Agreement	Threshold agreement achieved
		b) 50-80%,<br>c) >80%, and<br>d) Don't know				
Patient adherence (Is supervised treatment or any other interventions being implemented at a large scale to improve patient adherence to radical cure of vivax in your country?)	10.2a Do you think the question "Is supervised treatment or any other interventions being implemented at a large scale to improve patient adherence to radical cure of vivax in your country?" is adequate for capturing this factor?		19	18	94%	Yes
		10.2b We have classified implementation of supervised treatment or any other interventions into three categories as: a) Yes, b) No, c) Don't know	15	14	95%	Yes
Pharmacovigilance	11.a Do you think the question "What is the status of adverse event reporting for any disease in		15	12	80%	Yes

Factor	Question	Categorization	Total respondents who answered yes/no	Number of respondents who agree that the question/categorization adequately captures the factor	% Agreement	Threshold agreement achieved
	the last 12 months in your country?" is adequate for capturing this factor?					
		11.b We have classified Pharmacovigilance into four categories as: a) Adverse Event usually recorded and reported, b) Adverse Event sometimes recorded and reported, c) Adverse Event not recorded or reported, and d) Don't know	12	11	91%	Yes
Budget	12.a Do you think the question "What percentage of the annual budget for malaria is funded by the national government?" is adequate for capturing this factor?		17	13	76%	Yes
		12.b We have classified Budget as the percentage of the annual budget for malaria is funded by the national government.	11**	8	73%	No
Political will	13.a Do you think the question "Who was the chief guest in the last World Malaria Day event in your country?" is adequate for capturing this factor?		9**	6	67%	No
		13.b We have classified Political will with the proxy of the highest-level chief guest in the last	6**	6	100%	No

Factor	Question	Categorization	Total respondents who answered yes/no	Number of respondents who agree that the question/categorization adequately captures the factor	% Agreement	Threshold agreement achieved
		World Malaria Day event in your country as a) Prime Minister, b) Health Minister, c) Health/Permanent Secretary, d) Director General, e) Director of Department, or f) Others (specify)				
Risk aversion	14.a Do you think the question "What percentage of time was spent discussing patient safety compared to efficacy and implementation issues of 8-aminoquinolines in the last Technical Working Group (TWG) meeting which discussed on treatment policy change for vivax malaria in your country?" is adequate for capturing this factor?		15	14	93%	Yes
		14.b We have classified Risk aversion with the proxy of the percentage of time spent discussing "patient safety" compared to "efficacy" and "implementation issues of 8-aminoquinolines" in the country's last Technical Working Group (TWG) meeting which discussed on treatment policy change for vivax malaria.	14	14	100%	Yes

**Consensus not reached due to a wide variation*

***Minimum 12 respondents not reached*
