# Supplementary material for: Optimizing test and treat options for vivax malaria: An options assessment toolkit (OAT) for Asia Pacific national malaria control programs
Source: PLOS Glob Public Health. 2024 May 22;4(5):e0002970. doi: 10.1371/journal.pgph.0002970 (PMC11111040; doi:10.1371/journal.pgph.0002970)
Supplement: S10 Table — (PDF) [file pgph.0002970.s010.pdf]

S10 Table: Responses on the categorizations in each factor/ additional factor BAT (Round two Delphi)

Factor	Categorization	Total respondents who answered yes/no	Number of respondents who agree that the question/categorization adequately captures the factor	% Agreement	Threshold agreement achieved
Severity of G6PD deficiency	We have classified the Severity of G6PD deficiency into 4 categories, adapted from Malaria Policy Advisory Group, WHO 2022 as:<br>1. Class A: <20% median activity with chronic non-spherocytic hemolytic anemia (CNSHA)<br>2. Class B: <45% median activity with triggered acute hemolytic anemia (AHA)<br>3. Class C: 60-150% median activity without hemolytic risk<br>4. Class U: Any variant with unknown clinical significance and median activity	8*	7	87%	No
Budget	12.b We have classified Budget as the percentage of the annual budget for malaria is funded by the national government.	14	5	36%	No

**Minimum 12 respondents not reached*
