# Supplementary material for: Optimizing test and treat options for vivax malaria: An options assessment toolkit (OAT) for Asia Pacific national malaria control programs
Source: PLOS Glob Public Health. 2024 May 22;4(5):e0002970. doi: 10.1371/journal.pgph.0002970 (PMC11111040; doi:10.1371/journal.pgph.0002970)
Supplement: S11 Table — (PDF) [file pgph.0002970.s011.pdf]

S11 Table: Overview of scenarios using a simplified version of BAT

**Scenario 1**<br><br>(***Best case scenario***)	**Scenario 2**<br><br>(***At the finish line***)	**Scenario 3**<br><br>(***Running***)	**Scenario 4**<br><br>(***Walking***)	**Scenario 5**<br><br>(***Worst case scenario/sleeping***)
• No indigenous vivax cases	• Low vivax caseload<br>• Efficacy for low-dose PQ evident	• Low vivax caseload<br>• Efficacy for low-dose PQ evident	• High vivax caseload<br>• Efficacy for high dose PQ	• High vivax caseload<br>• No data on Efficacy for PQ
• Strong health system readiness	• Strong health system readiness	• Weak health system readiness	• Weak health system readiness	• No data/ Weak health system readiness
• Strong high-level political will	• Low risk aversion<br>• Strong high-level political will	• Low risk aversion<br>• Strong high level political will	• High risk aversion<br>• Strong high level political will	• Low/ High risk aversion<br>• Weak high level political will
