# Supplementary material for: Optimizing test and treat options for vivax malaria: An options assessment toolkit (OAT) for Asia Pacific national malaria control programs
Source: PLOS Glob Public Health. 2024 May 22;4(5):e0002970. doi: 10.1371/journal.pgph.0002970 (PMC11111040; doi:10.1371/journal.pgph.0002970)
Supplement: S12 Table — (PDF) [file pgph.0002970.s012.pdf]

S12 Table: Overview of 11 scenarios

	THEUNA	FLOESAL	CREOSO	OTROS	ACRINES	JOBLIL	PLOJI	GLAERA	ECHA	USPOS	BLAOR
**Enabling domain**											
**Budget (domestic funding)**	Moderate to High	Moderate to High	Low to Moderate	Moderate	Moderate	High	Low	Low to Moderate	Low to Moderate	Low	Low
**Political will**	High	High	Moderate to High	Moderate to High	Moderate to High	Moderate	Low to moderate	Moderate	Low	Low	Low
**Risk aversion of decision makers for future malaria policy options**	Moderate	Moderate	Moderate	low	Low	Moderate	High	**High**	Moderate - High	Low-Moderate	Low
**Implementation domain**											
**Referral initiation rate**	High	High	Very low	Moderate	Low to Moderate	Moderate	Low	Very low or don't know	Very low or don't know	Very low or don't know	Don't know
**Referral completion rate**	High	High	High	High	High	Moderate	Low	Very low or don't know	Very low or don't know	Very low or don't know	Don't know
**HW availability: community-level case management**	HW can test and track but cannot treat	HW can test and track but cannot treat	HW can test and track but cannot treat	HW can test and track but cannot treat	HW can test track but cannot treat	HW can test, treat, and track patient adherence	HW can test and track but cannot treat	HW can test and track but cannot treat	HW can test and track but cannot treat	HW can test and track but cannot treat	HW can test and track but cannot treat

	THEUNA	FLOESAL	CREOSO	OTROS	ACRINES	JOBLIL	PLOJI	GLAERA	ECHA	USPOS	BLAOR
**HW compliance with protocols**	High	High	Moderate to High	Moderate to High	Moderate to High	Moderate to High	Low or don't know	Low or don't know	Low or don't know	low	Don't know
**Supervised treatment for patient adherence**	Yes	Yes	Yes	Yes	Yes/No	Yes/No	Yes/No	No	No	No	No
**Patient adherence rate**	High	High	Moderate to High	Low to Moderate	Moderate	Moderate	Low or don't know	Low or don't know	Low or don't know	No	No
**Pharmacovigilance**	High	High	Moderate	Moderate to High	Moderate to High	Low to Moderate	Low	Low	Low	Low or don't know	Don't know
**Epidemiological domain**											
**Malaria program phase**	Prevention of re-introduction & Elimination	Prevention of re-introduction with outbreaks	Elimination	Elimination	Elimination	Elimination	Pre-elimination	Control	Control	Control	control
**Vivax caseload**	0	1-10,000	1-10,000	1-10,000	1-10,000	1-10,000	>10,000	>10,000	>10,000	>10,000	>10,000
**G6PD Def. Prevalence**	Common	common	Common	Common	**High**	Rare	Common–High	Common–High	Common - High	Common - High	Don't know
**Most common G6PD variants**	Kaiping / Canton/ don't know	Kaiping/ Canton/ don't know	Mediterranean/ Orissa/ don't know	Union/ Viangchan	Mahidol/ Viangchan	Don't know	Mahidol/ Viangchan	Viangchan/ Union/Vanua Lava	Mediterranean, Orissa, Kerala-Kalyan	Don't know	Don't know

	THEUNA	FLOESAL	CREOSO	OTROS	ACRINES	JOBLIL	PLOJI	GLAERA	ECHA	USPOS	BLAOR
**Current liver-stage treatment**	PQ14 (3.5mg/kg)/ PQ8Wk	PQ14 (3.5mg/kg)/ PQ8Wk	PQ14 (3.5mg/kg) / PQ8Wk	PQ14 (3.5mg/kg)/ PQ8Wk	PQ14 (3.5mg/kg) / PQ8Wk	PQ14 (3.5mg/kg) / PQ8Wk	PQ14 (3.5mg/kg)/ PQ8Wk	PQ14 (3.5mg/kg) / PQ8Wk	PQ14 (3.5mg/kg) /PQ8Wk	PQ14 (3.5mg/kg) /PQ8Wk	PQ14 (3.5mg/kg) / PQ8Wk
**Anti-relapse efficacy data**	No data available	No data available	Adequate (Risk of recurrence at 6 months at 1%, but >10% at 1 year)	Adequate (The risk of recurrence at 6 months around 10%)	**Inadequate** (The risk of recurrence at 6 months around 20%)	Inadequate (The risk of recurrence at 6 months around 20%)	Adequate (The risk of recurrence at 6 months around 10%)	**Inadequate** (The risk of recurrence at 6 months around 40%)	Adequate (The risk of recurrence at 6 months around 10%, but >10% at 1 year)	Inadequate (The risk of recurrence at 6 months around 40%)	No data available
**Delphi first round top options matching** **(out of 12 questions)**	100% match with Floesal	100% match with Theuna	100% match with Otros	100% match with Creoso	83% (i.e.10/12) match with Otros, Creoso, and Joblil	83% (i.e.10/12) match with Otros, Creoso, and Acrines	75% (i.e. 9/12) match with Glaera 66% (8/12) match with Joblil	75% (i.e. 9/12) match with Ploji 66% (8/12) match with Echa	91% (11/12) match with Uspos. 100% match with Blaor	91% (11/12) match with Echa and Blaor	91% (11/12) match with Uspos. 100% match with Echa
