# Supplementary material for: Optimizing test and treat options for vivax malaria: An options assessment toolkit (OAT) for Asia Pacific national malaria control programs
Source: PLOS Glob Public Health. 2024 May 22;4(5):e0002970. doi: 10.1371/journal.pgph.0002970 (PMC11111040; doi:10.1371/journal.pgph.0002970)
Supplement: S13 Table — (PDF) [file pgph.0002970.s013.pdf]

S13 Table: Scenario-based test and treat options (expert agreement on responses from Delphi- Qualitative and quantitative G6PD testing)

Scenario	Optimal G6PD test	Liver-stage treatment option/s			
		With **CQ** as the blood-stage treatment		With **ACT** as the blood-stage treatment	
		**For G6PD Normal (% of experts)**	**For G6PD intermediate (% of experts)**	**For G6PD Normal (% of experts)**	**For G6PD intermediate (% of experts)**
1. Theuna-Floesal	Point of care Quantitative test for G6PD	TQ (74%)	PQ14 (low dose) (74%)	PQ7 (high dose) (84%)	PQ14 (low dose) (68%)
		PQ7 (high dose) (26%)	PQ7 (low dose) (26%)	PQ14 (high dose) (16%)	PQ7 (low dose) (32%)
2. Creoso-Ortos	Point of care Quantitative test for G6PD	TQ (89%)	PQ14 (low dose) (68%)	PQ7 (high dose) (84%)	PQ14 (low dose) (68%)
		PQ7 (high dose) (11%)	PQ14 (high dose) (32%)	PQ14 (high dose) (16%)	PQ7 (low dose) (32%)
3. Acrines	Point of care Quantitative test for G6PD	TQ (84%)	PQ14 (low dose) (68%)	PQ7 (high dose) (74%)	PQ14 (low dose) (58%)
		PQ14 (high dose) (16%)	PQ14 (high dose) (32%)	PQ14 (high dose) (26%)	PQ14 (high dose) (42%)
4. Joblil	Point of care Quantitative test for G6PD	TQ (95%)	PQ14 (low dose)(53%)	PQ7 (high dose) *(84%)	PQ14 (low dose) (63%)
		PQ7 (high dose) (5%)	PQ7 (low dose) (42%)	PQ14 (high dose) (16%)	PQ7 (low dose) (37%)

5.	Ploji	Point of care Qualitative test for G6PD	TQ (68%)	PQ14 (low dose) (21%)	PQ7 (high dose) *(79%)	PQ14 (low dose) (68%)
			PQ14 (low dose) (21%)	PQ7 (low dose) (31%)	PQ14 (low dose) (21%)	PQ7 (low dose) (32%)
6.	Glaera	Point of care Quantitative test for G6PD	TQ (89%)	PQ7 (low dose) (89 %)	PQ7 (high dose) *(84%)	PQ14 (low dose) (53%)
			PQ7 (high dose) (11%)	PQ7(high dose) (11%)	PQ14 (low dose) (16%)	PQ7 (low dose) (47%)
7.	Echa-Blaor	Point of care Qualitative test for G6PD	PQ7 (high dose) *(84%)	PQ14 (low dose) (68%)	PQ7 (high dose) *(74%)	PQ14 (low dose) (74%)
			PQ14 (low dose) (16%)	PQ7 (high dose) (32%)	PQ14 (low dose) (26%)	PQ7 (low dose) (26%)
8.	Uspos	Point of care Qualitative test for G6PD	TQ (68%)	PQ7 (low dose) (53%)	PQ7 (high dose) * (84%)	PQ14 (low dose) (79%)
			PQ7 (high dose) (32%)	PQ14 (low dose) (37%)	PQ14 (high dose) (16%)	PQ14 (high dose) (21%)

**Replaced by PQ14 (high dose) in G6PD normal patients when presented to NMCP at the annual meeting in Dec 2022, given the issued WHO recommendation against high dose PQ7 in Nov 2022*
