# Supplementary material for: Optimizing test and treat options for vivax malaria: An options assessment toolkit (OAT) for Asia Pacific national malaria control programs
Source: PLOS Glob Public Health. 2024 May 22;4(5):e0002970. doi: 10.1371/journal.pgph.0002970 (PMC11111040; doi:10.1371/journal.pgph.0002970)
Supplement: S1 Fig — (PDF) [file pgph.0002970.s014.pdf]

**S1 Fig. Scenario CREOSO-OTROS.**

**Epidemiological factors:**

**Malaria program phase**: The Creoso/Otros countries are in the Elimination phase with <1 case/1000 population at risk/year.

**Vivax caseload**: The countries report vivax cases ranging from 1-10,000 per annum.

**G6PD deficiency prevalence**: The G6PD deficiency prevalence is estimated as common (1-10%).

**Liver stage treatment:** The recommended current radical cure regime is PQ at a low dose (3.5mg/kg total dose) given over 14 days or a weekly dose (0.75mg/kg) for 8 weeks.

**Antirelapse efficacy**: The efficacy of PQ14 low dose is estimated as adequate. The risk of recurrence of the current PQ 14 day treatment is estimated to be around 1-10% at 6 months.

**Implementation factors:**

**Referral initiation rate**: The proportion of vivax patients who get referred to a higher-level health facility after getting diagnosed at the community level can vary from very low (<10%) to moderate (>50-80%).

**Referral completion rate:** A high proportion of referred vivax patients (i.e., >80%) avail treatment at a higher-level facility.

**Community level case management:** There are health workers in the community who can test to confirm malaria and track but cannot treat.

**Health worker compliance rate:** A moderate (50-80%) to high (>80%) proportion of health workers are estimated to comply with treatment protocols.

**Patient adherence rate**: The proportion of vivax patients who adhere to recommended radical cure can vary from low (<50%) to high (>80%).

**Interventions to improve patient adherence:** The MOH in Creoso/Otros admits vivax malaria patients to hospital to provide DOT or provide supervised treatment like scheduled follow-up by community to ensure adherence to the treatment.

**Pharmacovigilance**: The pharmacovigilance system has moderate to high capacity. Adverse events are sometimes or usually recorded and reported from health facilities to the national level.

**Enabling factors:**

**Budget:** The proportion of NMP activities that are funded domestically ranges from low (≤30%) to moderate (31-89%) with external technical assistance available from the donor agencies.

**Political will:** The country has a moderate to high political will to achieve elimination. A Health/Permanent Secretary or a head of state like the Prime Minister attends the ‘World Malaria Day’ event in advocacy and commitment to sustain the achievements made.

**Risk aversion of decision makers for future malaria policy options:** The Ministry of Health and National Malaria Program have low to moderate risk aversion. During NMPs Technical Working Group (TWG) meetings, less or equal time is spent discussing ‘patient safety’ compared to ‘efficacy’ and ‘implementation issues of 8-aminoquinolines’.
