# Supplementary material for: Optimizing test and treat options for vivax malaria: An options assessment toolkit (OAT) for Asia Pacific national malaria control programs
Source: PLOS Glob Public Health. 2024 May 22;4(5):e0002970. doi: 10.1371/journal.pgph.0002970 (PMC11111040; doi:10.1371/journal.pgph.0002970)
Supplement: S2 Fig — (PDF) [file pgph.0002970.s015.pdf]

S2 Fig. Scenario ACRINES.

Epidemiological factors:

Malaria program phase: The Acrines countries are in the elimination phase with <1 case/1000 population at risk/year.

Vivax caseload: The countries report vivax cases ranging from 1-10,000 per annum.

G6PD deficiency prevalence: The G6PD deficiency prevalence is estimated as high (>10%).

Liver stage treatment: The recommended current radical cure regime is PQ at a low dose (3.5mg/kg total dose) given over 14 days or weekly dose (0.75mg/kg) for 8 weeks.

Antirelapse efficacy: The efficacy of PQ14 low dose is estimated as inadequate. The risk of recurrence of the current PQ 14 day treatment at 6 months is estimated around 20%.

Implementation factors:

Referral initiation rate: Low (10-50%) to moderate (>50-80%) proportion of vivax patients get referred to a higher-level health facility after getting diagnosed at the community level.

Referral completion rate: A high proportion of referred vivax patients (i.e., >80%) avail treatment at a higher-level facility.

Community level case management: There are health workers in the community who can test to confirm malaria and track but cannot treat.

Health worker compliance rate: A moderate (50-80%) to high (>80%) proportion of health workers are estimated to comply with treatment protocols

Patient adherence rate: Adherence to radical cure is moderate (50-80%).

Interventions to improve patient adherence: Acrines may provide supervised treatment like the scheduled follow-up to ensure adherence to the treatment or supervised treatment does not exist.

Pharmacovigilance: The pharmacovigilance system has moderate to high capacity. Adverse events are sometimes or usually recorded and reported from health facilities to the national level.

Enabling factors:

Budget: The proportion of NMP activities that are funded domestically is moderate (31-89%) with external technical assistance available from the donor agencies.

Political will: There is moderate to high political will to sustain the elimination. A Health/Permanent Secretary or a head of state like the Prime Minister attends the ‘World Malaria Day’ event in advocacy and commitment to sustain the achievements made.

Risk aversion of decision makers for future malaria policy options: Risk aversion is low. During NMPs Technical Working Group (TWG) meetings, less time is spent thinking through ‘patient safety’ compared to ‘efficacy’ and ‘implementation issues of 8-aminoquinolines’.
