# Supplementary material for: Optimizing test and treat options for vivax malaria: An options assessment toolkit (OAT) for Asia Pacific national malaria control programs
Source: PLOS Glob Public Health. 2024 May 22;4(5):e0002970. doi: 10.1371/journal.pgph.0002970 (PMC11111040; doi:10.1371/journal.pgph.0002970)
Supplement: S3 Fig — (PDF) [file pgph.0002970.s016.pdf]

S3 Fig. Scenario JOBLIL.

Epidemiological factors:

Malaria program phase: The countries in Joblil are in the elimination phase, defined as <1 case/1,000 populations at risk per year.

Vivax caseload: The countries are characterized by an annual caseload of vivax ranging from 1-10,000.

G6PD deficiency prevalence: The G6PD deficiency prevalence is estimated as rare (<1%).

Liver stage treatment: The recommended current radical cure regime is PQ at a low dose (3.5mg/kg total dose) given over 14 days or weekly dose (0.75mg/kg) for 8 weeks.

Antirelapse efficacy: The efficacy of the current PQ14 treatment is unknown. However, regionally, it is estimated that risk of recurrence at 6 months is 20%.

Implementation factors:

Referral initiation rate: A moderate proportion of vivax patients (i.e., 50-80%) get referred to a higher-level health facility after getting diagnosed at the community level.

Referral completion rate: Moderate proportion of referred vivax patients (i.e., 50-80%) avail treatment at a higher-level facility.

Community level case management: There are health workers at the community level that can test to confirm malaria treat and track patients for adherence.

Health worker compliance rate: A moderate (50-80%) to high (>80%) proportion of health workers are estimated to comply with treatment protocols.

Patient adherence rate: Adherence to radical cure is moderate (50-80%).

Interventions to improve patient adherence: Joblil may provide supervised treatment like a scheduled follow-up to ensure adherence to the treatment or supervised treatment does not exist.

Pharmacovigilance: The pharmacovigilance system has low to moderate capacity. Adverse events are either not or only sometimes recorded and reported from health facilities to the national level.

Enabling factors:

Budget: The proportion of NMP activities that are funded domestically is high (≥ 90%). However, remaining gaps in funds along with external technical assistance are available from the donor agencies

Political will: The country has a moderate political will to progress to elimination. The Health/Permanent Secretary attends the ‘World Malaria Day’ event in advocacy and commitment to sustain the achievements made.

Risk aversion of decision makers for future malaria policy options: Risk aversion is moderate. During NMPs Technical Working Group (TWG) meetings equal time is spent discussing ‘patient safety’ as it is for ‘efficacy’ and ‘implementation issues of 8-aminoquinolines’.
