# Supplementary material for: Optimizing test and treat options for vivax malaria: An options assessment toolkit (OAT) for Asia Pacific national malaria control programs
Source: PLOS Glob Public Health. 2024 May 22;4(5):e0002970. doi: 10.1371/journal.pgph.0002970 (PMC11111040; doi:10.1371/journal.pgph.0002970)
Supplement: S5 Fig — (PDF) [file pgph.0002970.s018.pdf]

S5 Fig. Scenario GLAERA.

Epidemiological factors:

Malaria program phase: The countries in Glaera are in control phase, defined by slide or RDT positivity rate ≥ 5%.

Vivax caseload: The countries are characterized by an annual vivax caseload of >10,000.

G6PD deficiency prevalence: The G6PD deficiency prevalence is estimated as common (1-10%) to high (>10%).

Liver stage treatment: The recommended current radical cure regime is PQ at a low dose (3.5mg/kg total dose) given over 14 days or weekly dose (0.75mg/kg) for 8 weeks.

Antirelapse efficacy: The estimated efficacy of the current PQ14 treatment is inadequate. The risk of recurrence at 6 months is 40%.

Implementation factors:

Referral initiation rate: Very low proportion of vivax patients (i.e., <10%) get referred to a higher-level health facility after getting diagnosed at the community level or data is not available for initiation of referral.

Referral completion rate: Very low proportion of referred vivax patients (i.e., <10%) avail treatment at a higher-level facility, or data is not available for completion of referral.

Community level case management: Health workers at the community level can test to confirm malaria and track but cannot treat cases.

Health worker compliance rate: A low proportion of health workers (i.e., <50%) are estimated to comply with national malaria treatment protocols or data on their compliance rate is not available.

Patient adherence rate: Adherence to radial cure is low (<50%).

Interventions to improve patient adherence: No supervision of treatment or other interventions to improve patient adherence are implemented.

Pharmacovigilance: The pharmacovigilance system has low capacity. Adverse events not recorded and reported from health facilities to the national level.

Enabling factors:

Budget: The proportion of NMP activities that are funded domestically is low (≤30%) to moderate (31-89%). The remaining gaps in funds along with external technical assistance are available from the donor agencies.

Political will: The country has a moderate political will to progress to elimination. The Health/Permanent Secretary attends the ‘World malaria Day’ event in advocacy and commitment to sustain the achievements made.

Risk aversion of decision makers for future malaria policy options: Risk aversion is High. During NMPs Technical Working Group (TWG) meetings, more time is spent discussing ‘patient safety’ than ‘efficacy’ and ‘implementation issues of 8-aminoquinolines’.
