# Supplementary material for: Optimizing test and treat options for vivax malaria: An options assessment toolkit (OAT) for Asia Pacific national malaria control programs
Source: PLOS Glob Public Health. 2024 May 22;4(5):e0002970. doi: 10.1371/journal.pgph.0002970 (PMC11111040; doi:10.1371/journal.pgph.0002970)
Supplement: S6 Fig — (PDF) [file pgph.0002970.s019.pdf]

S6 Fig. Scenario ECHA-BLAOR.

Epidemiological factors:

Malaria program phase: Echa/Blaor countries are in the control phase, defined by slide or RDT positivity rate ≥ 5%.

Vivax caseload: The countries are characterized by an annual vivax caseload of >10,000.

G6PD deficiency prevalence: The G6PD deficiency prevalence is estimated as common (1-10%) to high (>10%) or the data may not be available.

Liver stage treatment: The recommended current radical cure regime is PQ at a low dose (3.5mg/kg total dose) given over 14 days or weekly dose (0.75mg/kg) for 8 weeks.

Antirelapse efficacy: The estimated efficacy of the current PQ14 treatment is adequate. The risk of recurrence at 6 months is 1%, but >10% at 1 year. However, the data may not be available in some cases.

Implementation factors:

Referral initiation rate: Very low proportion of vivax patients (i.e., <10%) get referred to a higher-level health facility after getting diagnosed at the community level or data is not available for initiation of referral.

Referral completion rate: Very low proportion of referred vivax patients (i.e., <10%) avail treatment at a higher-level facility, or data is not available for completion of referral.

Community level case management: Health workers at the community level that can test to confirm malaria and track but cannot treat cases.

Health worker compliance rate: A low proportion of health workers (i.e., <50%) are estimated to comply with national malaria treatment protocols or data on their compliance rate is not available.

Patient adherence: Adherence to radial cure is low (<50%) or data may not be available.

Interventions to improve patient adherence: No supervision of treatment or other interventions to improve patient adherence are implemented.

Pharmacovigilance: The pharmacovigilance system has low capacity. Adverse events not recorded and reported from health facilities to the national level.

Enabling factors:

Budget: The proportion of NMP activities that are funded domestically is low (≤30%) to moderate (31-89%). Remaining gaps in funds along with external technical assistance are available from the donor agencies

Political will: The country has a low political will to sustain progress to elimination. No High-ranking official attends the ‘World Malaria Day’ event in advocacy and commitment to sustain the achievements made.

Risk aversion of decision makers for future malaria policy options: Risk aversion among the Ministry of Health and National Malaria Program can vary from moderate to high, or it cannot be ascertained. During NMPs Technical Working Group (TWG) meetings, equal or more time is spent discussing ‘patient safety’ compared to ‘efficacy’ and ‘implementation issues of 8-aminoquinolines’ or TWG meetings are held sporadically.
