# Supplementary material for: Optimizing test and treat options for vivax malaria: An options assessment toolkit (OAT) for Asia Pacific national malaria control programs
Source: PLOS Glob Public Health. 2024 May 22;4(5):e0002970. doi: 10.1371/journal.pgph.0002970 (PMC11111040; doi:10.1371/journal.pgph.0002970)
Supplement: S1 Text — (PDF) [file pgph.0002970.s021.pdf]

Approaches tool for OAT

Objective:

- To outline policy change process considerations and highlight different implementation strategies/approaches for each combination of radical cure options.
- To plan the next steps to use OAT beyond 2022/23

Description

This group work should enable the NMPs to visualize the policy change process and provide their own recommendations for the effective implementation of the chosen test and treatment combination. Specific approaches will include strategies for targeted allocation of resources, tests and treatments, improving access, adherence, advocacy, and awareness for the radical cure combinations.

WHEN should this consideration be used?

The consideration for policy change and approaches to implement more effective test and treat combinations for vivax malaria must be made if country has been missing out the target for malaria elimination due to vivax malaria.

WHO should be responsible for this process?

The NMP should take the lead role. After deciding the optimal radical cure option, the TWG and NMP can draw valuable insights from this tool to proceed and plan to reach the 2030 malaria elimination target.

WHY was there a need for this consideration for policy change?

While some countries may have an existing systemic approach to tackle policy changes routinely, some countries may lack such routine approaches, and therefore, they may not understand the processes and approaches involved.

Policy change may take very long in some countries, but with the availability of evidence for policy decisions, such processes can be fast-tracked. Toward the 2030 malaria elimination target, there is a need to fast-track the management process of vivax malaria.

HOW should it be done?

The NMPs should identify the need for changing the vivax clinical management based on the scenario. NMP and TWG should access expert technical guidance on test and treat policy and adapt/develop clinical

management guidelines. Then, the clinical guidelines should be put up with the relevant authority for consideration for policy change and, finally, approval.

Considerations for policy change and implementation of vivax radical cure

Policy changes processes:

- **Who do you need to convene to change policy to the selected test and treat options**?
  - ~ What committees are normally convened to consider a policy change? In some countries, this might include a Technical Working Group and National Drug Committee.
  - ~ How often does that group/committee meet to review new test and treatment options? When is the next meeting?
  - ~ What other processes need to be considered (e.g., application for Essential Medicines List)? Are there pilot study requirements? Are those required before or after the test and drug registration?
  - ~ Identify where you think you may need to undertake advocacy for policy change and resources to support implementation (e.g., immigration dept, HMIS, pharmacovigilance units)
  - ~ Consider making adapted versions of the evidence summaries and registration updates that were provided during earlier parts of the meeting. Consider making country experiences available to key decision-makers (e.g., through the technical working group)

Implementation factors

Targeting & access

- ~ Has your program undertaken sub-national tailoring or stratification?
- ~ If yes:
  - o When was it undertaken most recently?
  - o Is it feasible to allocate test and treatment options chosen *initially* in the high-burden strata identified through SNT or stratification?
  - o From the access session in which we discussed ways to achieve access – please consider which options discussed may have been most feasible for your context,

whether a mix of strategies is required or how you think it is best to achieve access where vivax caseloads are highest?

- Think about access to remote, mobile or border populations – is a system such as ‘buddy health’ feasible for your setting?

~ If no, consider the following:

- Criteria from FIND on where to place G6PD analyzers.
