# Supplementary material for: Optimizing test and treat options for vivax malaria: An options assessment toolkit (OAT) for Asia Pacific national malaria control programs
Source: PLOS Glob Public Health. 2024 May 22;4(5):e0002970. doi: 10.1371/journal.pgph.0002970 (PMC11111040; doi:10.1371/journal.pgph.0002970)
Supplement: S2 Text — (PDF) [file pgph.0002970.s022.pdf]

<u>Step-by-step guide on using the OAT</u>

Introduction

The vision of WHO and the global malaria community is a world free of malaria. As part of this vision, the Global Malaria Technical Strategy 2018-2030 set ambitious global targets for 2030. Countries that have controlled malaria *Plasmodium falciparum* malaria cases are reducing, but as this happens, the proportion of cases due to *Plasmodium vivax* has increased. *P. vivax* tolerates a wider range of environmental conditions than *P. falciparum* and, therefore, has a wider geographical range. In countries where both *Plasmodium falciparum* and *P. vivax* are present, the disease burden due to *P. vivax* is more difficult to reduce because the parasite forms a dormant stage in the liver (hypnozoites). Dormant hypnozoites are more difficult to detect because the parasite density is typically low, and dormant hypnozoites residing in the liver cannot be detected with existing diagnostic tests. Hypnozoites can give rise to multiple relapses and contribute to significant morbidity and onward transmission. *P. vivax* can be transmitted from humans to mosquitoes before infected people develop symptoms. The hypnozoites can only be eliminated through treatment with drugs belonging to the 8-aminoquinoline class, which can produce serious side effects (hemolytic anemia) in patients who have G6PD deficiency, and such treatment is contraindicated in vulnerable population groups such as infants and pregnant or breastfeeding women.

WHO recognizes that safe and effective radical treatment of vivax malaria currently requires two diagnoses (confirmation of *P. vivax* parasites and glucose-6-phosphate dehydrogenase (G6PD) status). As point-of-care G6PD tests become available, these services will need to be implemented alongside malaria diagnostic testing to ensure optimal treatment to prevent *P. vivax* relapse. For elimination to succeed, greater attention must be given to *P. vivax,* a parasite less well-understood than P. *falciparum*. Vivax malaria presents multiple challenges and needs specific strategies.

The control and elimination of malaria depend on a resolute political commitment to universal health care, inclusive of malaria prevention, diagnosis, and treatment as part of both primary healthcare systems and broader development initiatives.

Human and financial resources will be required to appropriately support safe and effective implementation of G6PD testing services and improved radical treatment of vivax malaria. The services, both public and private, and medical products need to be safe and effective and delivered in a timely, equitable, efficient and integrated manner. High-quality and integrated delivery are important for reducing both the burden of malaria and the potential for onward transmission of parasites.

To keep ahead of the disease will require a culture of learning and adapting with the capacity to effectively generate and use knowledge to identify gaps, health disparities and existing inequalities, monitor progress, and seek and adopt transformative approaches and new interventions that have the potential to accelerate the progress towards elimination goal of 2030.

Background and rationale

As countries in the Asia Pacific strive towards the elimination of malaria by 2030, many may have eliminated P. falciparum. To achieve elimination, attention must be given to P. vivax the treatment of which requires a cure for both blood- and liver (hypnozoite)- stages of the disease.

Currently, primaquine is the only widely available and WHO-recommended drug against hypnozoites. However, the prolonged administration of PQ for 14 days or 8 weeks brings means patient adherence is a risk leading to a failure to

complete the recommended treatment regimen. There is also a risk of hemolysis from 8-aminoquinolines for patients with less than normal G6PD activity. Recent advances in near-patient or point-of-care G6PD deficiency screening and shorter course 8-aminoquinoline treatments are rapidly changing the landscape of radical cure of vivax malaria available for National Malaria Programs (NMPs).

While NMPs await the WHO's global policy guidance on these advances, they need to consider different contextual factors related to their countries' vivax burden, health system capacity, and availability of resources to support changes to their policies and practices.

This Options Assessment Toolkit (OAT) was developed to enable NMPs to determine optimal radical cure options for their given environments systematically. Where multiple options are available to policymakers, evidence shows that delays in decision-making occur. Malaria program policy changes are often triggered in reaction to WHO recommendations from the Global Malaria Program (see figure 1A). This more proactive approach using the OAT for decision-making and identifying activities to strengthen support mechanisms to facilitate the effective use of new tools can shorten delays once WHO recommendations are available from the global level and potentially reduce the size of stairs along the loss of effectiveness stairway.

The tools within OAT are designed to encourage discussion among NMPs about the best test and treatment approaches for their given contexts.

The OAT has been co-developed through a participatory research methods approach, which included validation of the various elements with NMPs and experts engaged in designing the research process and the toolkit.

Figure 1: Conceptual timelines for reactive (A) and proactive (B) approaches to decision-making

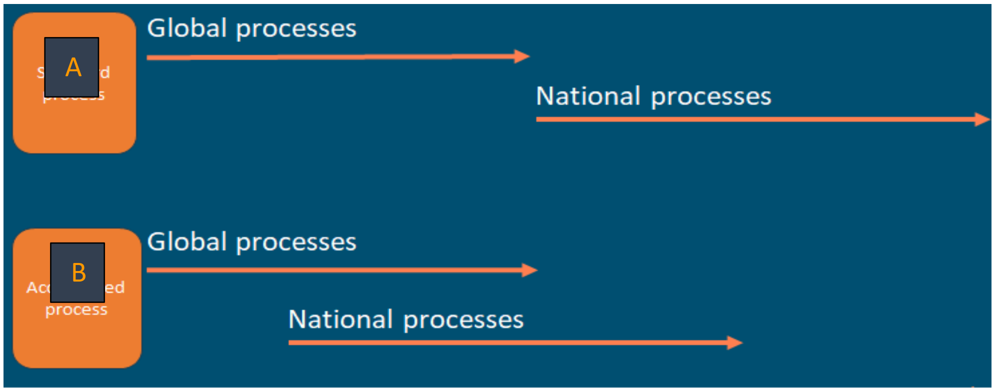

What is OAT and why do it?

The OAT kit consists of:

1) Baseline Assessment Template (BAT)
2) Scenarios representing the Asia Pacific region
3) Scenario-based test and treat options
4) Considerations for policy change and approaches to implement Test and Treat combinations
5) Step-by-step guide

These tools are expected to be used by the NMPs and their stakeholders working in antimalarial response in the country/region and facilitate accelerated decision-making for an optimal radical cure for vivax malaria and accelerate towards achieving malaria elimination.

Table 1: OAT elements, description, objective, and intended use/user:

Tool	Description	Objective	Intended use/user
1. Baseline assessment template (BAT)	This template includes specific variables under three broad factors of epidemiological, implementation (health system), and enabling (political and economic) factors	Facilitate the NMPs in assessing their current situation of readiness for vivax elimination.	For use by the TWG at the start of the process for considering changing antimalarial policy or improving current vivax treatment guidelines.
2. Scenarios representing the Asia Pacific region	This tool describes eight possible contextual scenarios depicting the status of malaria elimination/control in the Asia Pacific region, taking into consideration the readiness of health system and politico-economic factors, along with vivax malaria-specific epidemiological factors.	Aid the NMPs in viewing different contextual and health system features of scenarios of malaria elimination in the region.	The NMP and TWG can map the range of scenarios possible in the region and identify which scenario their country likely falls into.
3. Scenario-based test and treat options	This document will list the expert-recommended optimal combinations of G6PD testing and radical cure treatment regimens for each scenario.	Enable the NMPs to assess the combinations of radical cure options for their current scenario.<br><br>Allow the NMPs to view what the experts think and visualize potential future scenarios and radical cure tool combinations as burden reduces in higher-burden countries.	The TWG to discuss in detail the options recommended by the experts, their feasibility for the local country context, and then identify what is optimal for their conditions
4. Considerations for policy change and approaches to implement Test and Treat combinations	This tool will enable the NMPs to visualize the policy change process and provide recommendations for effective implementation of the chosen test and treat combination. Specific approaches will include strategies on improving access, adherence, allocation of resources, advocacy, and awareness for the radical cure combinations.	Outline policy change process considerations and highlight different implementation strategies/approaches for each combination of radical cure options.	After deciding the optimal radical cure option, the TWG and NMP can draw valuable insights from this tool to proceed and plan for reaching the 2030 malaria elimination target.

OAT elements

1. Baseline Assessment Template (BAT)

Objective: To enable NMPs to assess the current readiness for vivax elimination in their country context.

Description: BAT provides a comprehensive framework of specific variables for assessing the enabling, implementing, and epidemiological factors and identifies gaps that need strengthening to improve/update testing and treatment for vivax malaria.

WHEN should this Baseline assessment template be used?
The baseline assessment template should be used when the NMP requires updating/revising the vivax malaria test and treatment policy and needs to know the current status so that they can choose from the various recommended tests and treatment options that best suit the local context.

WHO should do the baseline assessment?
The NMPs should lead the baseline assessments in each country and identify their stakeholders in the Ministry of Health (HMIS, Research Unit, AFD, PPD, District Health Services, Pharmacy Department Clinical Laboratory and Public Health Labs, etc.) and Allied Health Agencies (Medical Council, Drug Regulatory Authority, etc.) who can provide the information on various aspects of health financing and health system.

WHY was there a need for a baseline assessment template?
Countries in the Asia Pacific region are in different phases of malaria programming and have diverse challenges. A standard, tested, baseline assessment template (BAT) can enable NMPs to focus on critical information that needs to be considered while revising or updating the treatment policy for vivax malaria.

HOW should it be done?
NMPs should take the lead role in completing the baseline assessment template. Firstly, the NMPs should fill out all the information the program can confidently fill out. NMPs should identify and consult relevant stakeholders for relevant information (see Table 2). If there is already an existing committee that the program works with routinely, such committees should support the program. If there is no such committee, a committee with relevant stakeholders may be formed to complete the assessment. All information filled out should be backed up with evidence and references listed for authenticity. Where there is a lack of information, a study or research may be needed in the future if need be, so such gaps should also be identified and listed.

How long should this element take?
As per the experience shared by a few NMPs, the filling up of the BAT has just taken about 30-60 minutes to fill, but it may vary from country to country based on various factors.

2. Scenarios representing the Asia Pacific region

Objective: To aid the NMPs in viewing different contextual and health system features of scenarios of malaria elimination in the region.

Description: This tool describes eight possible contextual scenarios depicting the status of malaria elimination/control in the Asia Pacific region, considering the health system's readiness, politico-economic factors, and vivax malaria-specific epidemiological factors.

WHEN should this scenario be assessed?
This assessment should be useful when the NMP and TWG plan to revise /improve the test and treat options for vivax malaria.

WHO should do the scenario assessment?
The NMP and TWG can map the range of scenarios possible in the region and identify which scenario their country likely falls into.

WHY was there a need to know the scenario?
Knowing the scenario will help understand the country context and where they currently stand among the Asia Pacific countries, and based on the scenario, they can decide what are the most feasible test and treatment options for vivax malaria management.

HOW should it be done?
The Baseline Assessment Template should be completed properly through a consultative process by the NMPs, their stakeholders, and the Technical Working Group, using validated data as much as possible to capture the country context reasonably close to the actual scenario.

How long should this element take?
This is a critical step and will require a good amount of discussion and consensus among the NMP and stakeholders to arrive at the scenario that is most realistic and close to the actual scenario.

3. Scenario-based test and treat options

Objective: To enable the NMPs to assess the combinations of G6PD testing and radical cure options for their current scenario. Also, to allow the NMPs to view what the experts think and visualize potential future scenarios and radical cure tool combinations as burden reduces in higher-burden countries.

Description: This document will list the expert-suggested optimal combinations of G6PD testing and radical cure treatment regimens for each scenario.

WHEN should this tool be used?
These expert views should be used when NMPs plan to revise/improve their test and treatment policy for vivax malaria and also at a later stage when they are processing with the policymakers for approval of the new policy.

WHO should use the Scenario-based test and treat options?
The TWG should refer to this tool to discuss and decide the options recommended by the experts, their feasibility for the local country context, and then identify what is optimal for their conditions.

WHY was there a need for Scenario-based test and treat options?
This expert feedback on test and treatment options per scenario is needed as most countries may not have national experts to help in technical decision-making in vivax malaria case management based on local context. Without a set of broad overarching guidelines suggested by the experts, the countries may end up making all sorts of different tests and treatment options.

HOW should it be used?
Once the country has decided on the scenario, the feasible test and treatment options should be decided. However, other factors like enabling and political factors may need to be considered for final decision-making.

How long should this element take?
This policy decision-making may require a few meetings among the Technical Working Group members and at least one meeting at the policy level to approve in case additional resources (Financial and HR) is needed to implement the change.

4. Considerations for policy change and approaches to implement Test and Treat combinations

Objective: To outline policy change process considerations and highlight different implementation strategies/approaches for each combination of radical cure options.

Description: This tool will enable the NMPs to visualize the policy change process and provide recommendations for effective implementation of the chosen test and treat combination. Specific approaches will include strategies for improving access, adherence, allocation of resources, advocacy, and awareness for the radical cure combinations.

WHEN should this consideration be used?
The consideration for policy change and approaches to implement test and treatment combinations for vivax malaria must be made if a country has been missing out on the target for malaria elimination due to vivax malaria.

WHO should be responsible for this process?
The NMP should take the lead role. After deciding the optimal radical cure option, the TWG and NMP can draw valuable insights from this tool to proceed and plan to reach the 2030 malaria elimination target.

WHY was there a need for this consideration for policy change?
While some countries may have an existing systemic approach to tackle policy changes routinely, some countries may lack such routine approaches, and therefore, such countries may need to understand the processes and approaches involved. Policy changes may take very long in some countries, but with the availability of evidence for policy decisions, such processes can be fast-tracked. Toward the 2030 malaria elimination target, there is a need to fast-track the process of management of vivax malaria.

HOW should it be done?
The NMPs should identify the need to change the vivax clinical management based on the scenario. NMP and TWG should access Expert technical guidance on test and treat policy and adapt/develop clinical management guidelines. Then, the clinical guidelines should be put up to the relevant authority for policy change consideration and approval.

How long should this element take?
The process for policy change may take months to several years in some countries. It would depend on how the health systems function in each country.

Table 2: Baseline assessment template domains, questions, variables, stakeholders list and source of information (*each country may have different names for their committees)*

Domain	Questions in BAT	Variables	Stakeholders	Source of information
**Epidemiology**	What is the phase of malaria program in your country?	Prevention of re-introduction	Technical Working Group (or country equivalent)	National strategic plan, annual malaria report; world malaria report
		Elimination		
		Pre-elimination		
		Control		
	What is the number of annual reported cases of vivax in your country?	0	HMIS and WHO	Annual malaria program report; annual health bulletin; world malaria report
		1-10,000		
		>10,000		
	What is the level of G6PD deficiency (defined as less than 30% G6PD activity) in your country?	Rare (<1%)	Clinical laboratory/study report	Laboratory report; study report (national or local); collation of small-scale national studies, if no data available (Howes et al 2012)
		Common (1%-10%)		
		High (>10%)		
		Don't know		
	What is the anti-relapse efficacy of PQ14 low dose in your country or similar settings?	Adequate (>85% recurrence-free at six months)	Research unit/ethical committee	National or regional randomized controlled trial data; National Drug regulatory Authority such as FDA; and Ethical Committee where study protocols are submitted.
		Inadequate (<85% recurrence-free at six months)		
**Implementation**		High (>80%)	Health facility in-charge, data managers, researchers	Health facility referral registers report/study report
		Moderate (>50%-80%)		

Domain	Questions in BAT	Variables	Stakeholders	Source of information
	What is the estimated proportion of vivax patients referred from initial point of malaria diagnosis to higher health centers?	Low (>10%-50%)		
		Very low (<10%)		
		Do not know		
	What is the estimated proportion of referred vivax patients that complete referral at receiving health facility	High (>80%)	Health facility in-charge, data managers, researchers	Health facility report/study report (national or local)
		Moderate (>50%-80%)		
		Low (>10%-50%)		
		Very low (<10%)		
		Do not know		
	What activities are allowed by the Ministry of Health for health workers at the community level for malaria case management?	HW can test, treat, and track patient adherence	Medical council & drug regulatory authority	Malaria treatment guideline; medical council & drug regulatory authority regulations; labor force and employment surveys; health facility assessment and routine administrative information
		HW can test and track but cannot treat		
		HW available but cannot test, treat and track		
		Do not know.		
	What do you think is the estimated proportion of health workers at different levels of the health system who adhere to current or new treatment protocol?	High (>80%)	Pharmacy unit in health facilities	Pharmacy register, study report (national or local), routine case records
		Moderate (>50%-80%)		
		Low (<50%)		
		Don't know		
	What do you think is the estimated proportion of patients who adhere to the	High (>80%)	Research partners	National or local survey
		Moderate (>50%-80%)		

Domain	Questions in BAT	Variables	Stakeholders	Source of information
	full treatment regimen of current recommended radical cure of drugs?	Low (<50%)		
		Don't know		
	Is supervised treatment or any other intervention being implemented at a large scale to improve patient adherence to current recommended radical cure of vivax in your country? (Policy on supervised treatment)	Yes	Pharmacy unit in health facilities	National strategic plan, national treatment guidelines, pharmacy register, study report
		No		
		Don't know		
	What is the status of adverse event reporting for any diseases in the last 12 months in your country? (Pharmacovigilance)	High (AE usually recorded and reported from health facility to national level)	Pharmacy in health facility & drug regulatory authority	Report from drug regulatory authority pharmacovigilance unit (or national pharmacovigilance unit not linked with FDA)
		Moderate (AE sometimes recorded and reported health facility to national level)		
		Low (AE not recorded or reported health facility to national level)		
		Do not know		
**Enabling**	What Percentage of time was spent discussing "patient safety" compared to "efficacy" and implementation issues pf "8-aminoduinolines (PQ, TQ) in the last Technical Working Group (TWG) meeting which discussed on treatment	High (More time spent on discussing safety compared to efficacy)	TWG members	TWG meeting report
		Moderate (equal time spent on discussing safety compared to efficacy)		

Domain	Questions in BAT	Variables	Stakeholders	Source of information
	policy change for vivax malaria in your country?	Low (Less time spent on discussing safety compared to efficacy)		
	What percentage of the annual budget for malaria is funded by the national government?	High (≥90%)	PPD/AFD/MOH	Annual budget report
		Moderate (19-89%)		
		Low (≤20%)		
	Who was the chief guest in the last World Malaria Day event in your country?	High (Head of state attends World Malaria Day events)	NMP NMP	WMD report
		Moderate (Permanent Secretary attends World Malaria Day events)		
		Low (No high-ranking official attends World Malaria Day events)		
