# Supplementary material for: Optimizing test and treat options for vivax malaria: An options assessment toolkit (OAT) for Asia Pacific national malaria control programs
Source: PLOS Glob Public Health. 2024 May 22;4(5):e0002970. doi: 10.1371/journal.pgph.0002970 (PMC11111040; doi:10.1371/journal.pgph.0002970)
Supplement: Attachment — Submitted filename: Acharya_OATpaper_replies to reviewers.docx [file pgph.0002970.s023.docx]

We would like to thank the reviewers for the helpful and constructive feedback. We have now revised the manuscript in line with the comments and hope it will now meet PLOS Global Public Health’s publication criteria. Please see blow our point-by-point response.

Journal Requirements:

1. Please include a complete copy of PLOS’ questionnaire on inclusivity in global research in your revised manuscript. Our policy for research in this area aims to improve transparency in the reporting of research performed outside of researchers’ own country or community. The policy applies to researchers who have travelled to a different country to conduct research, research with Indigenous populations or their lands, and research on cultural artefacts. The questionnaire can also be requested at the journal’s discretion for any other submissions, even if these conditions are not met.  Please find more information on the policy and a link to download a blank copy of the questionnaire here: <https://journals.plos.org/globalpublichealth/s/best-practices-in-research-reporting>. Please upload a completed version of your questionnaire as Supporting Information when you resubmit your manuscript.

We have added the completed questionnaire as supporting information to the submission

2. Please ensure that Funding Information and Financial Disclosure Statement are matched.

Funding for this work was provided by MMV and APLMA through the VivAccess and APMEN grants. The work was supported, in whole or in part, by the Bill & Melinda Gates Foundation [OPP1194815 / INV-008202]. Under the grant conditions of the Foundation, a Creative Commons Attribution 4.0 Generic License has already been assigned to the Author Accepted Manuscript version that might arise from this submission. The funders had no role in the study design, data collection and analysis, decision to publish, or preparation of the manuscript.

3. Please provide separate figure files in .tif or .eps format only and remove any figures embedded in your manuscript file. Please also ensure all files are under our size limit of 10MB.

We have now provided those files.

4. We have noticed that you have uploaded Supporting Information files, but you have not included a list of legends. Please add a full list of legends for your Supporting Information files after the references list.

We have added this now.

Reviewers' comments:

Reviewer #1:

In this work, Acharya and colleagues describe a toolkit to support national malaria programs in the Asia Pacific region in the choice of their P. vivax radical cure strategy. The toolkit consists in a list of questions used to assess the situation and classify any given country into one of eight archetypical scenarios. Each scenario is then associated with a recommended radical cure policy. The elaboration of the toolkit was the result of consultations with NMP members and a panel of experts (protocol detailed in a previous publication). Although the toolkit was presented to relevant stakeholders, the authors indicate that the toolkit has not yet been utilized in a specific country context.
The paper is very interesting to read and it tackles an important topic, namely supporting decision making in a situation where the recommendations and types of diagnostics and treatments are difficult to navigate. I highly appreciate the inclusion of various stakeholders with diverse range of expertise and origin in the conception of the tool. Nonetheless, I have some major comments that I believe the authors should address for publication, as well as some minor remarks.

Major point
1- In my opinion, the link between the eight country scenarios and their associated recommended test and treat policy is unclear. The manuscript describes in details how these choices were made as a result of expert consensus, but does not justify why such policies are the most appropriate for each scenario given the current evidence. I believe such a justification should be included in the manuscript (and potentially the toolkit), so that the recommendations reflect the results of evidence-based decision making, beyond expert opinions. This is particularly important, especially as the authors mention that some recommendations from the experts are not aligned with those from the WHO (see discussion section).

*We made the assumption that experts in the field would be very familiar with the literature and the relevant evidence* *and that expert opinion is therefore* *evidence based*. *In this context there might be different interpretation about what the evidence suggest in terms of radical cure policies between the included expert and WHO. We have also highlighted that there is a lack of consensus to what constitutes an acceptable safety or tolerability. We have now expanded the discussion section to allude to this. It now reads as follows (line 355-360):*

*Experts selected PQ7 (high dose) for one scenario (with CQ as blood-stage treatment) and across all scenarios (with ACT as blood-stage treatment) when quantitative G6PD testing is available.This is in contrast to the 2022 WHO recommendations against PQ7 (high dose) citing which cites concerns over gastro-intestinal tolerability and increased risk for hemolysis (29). This discrepant interpretation of the available evidence is likely linked to varying levels of acceptance for risk and different sense of urgency (31).*

2- It seems that the test and treat recommendations for scenarios 1 to 5 are identical (and also scenarios 6 and 8). It would be useful if the authors could explain the need to have so many scenarios if they lead to the same policy.

*This is indeed true and was not an expected result. Our a priory assumption was that different scenarios would require different solutions. We have expanded the discussion section now to acknowledge this. It reads as follows (line 345-438)*

*“Vivax malaria in the Asia-Pacific region is heterogeneous and country contexts vary considerably. The a priory assumption in the development of the OAT was therefore that those different contexts matter for tailoring treatment policy. However the selected test and treat options varied little by scenario, suggesting that potentially a less differential approach to treatment policy is required.”*

3- I found it difficult to reconcile the objective of creating a user-friendly tool while stating that it should not be used as a prescriptive tool (in Discussion section: “a tool to guide discussion of and with decision makers, rather than as a tool kit where variables are entered and a specific output recommendation for optimal test and treatment options generated.”) It would be helpful if the authors could explain more precisely what makes their toolkit a support for discussion rather than a prescriptive tool.

*From the experience in the development process, discussion with the co-developing NMP representatives as well as the initial piloting we felt that the process of going through and discussing the different tools was appreciated and was a systematic way to support a decision making process*. *This is in contrast to a tool that merely spits out a certain recommendation that NMPs are supposed to follow.* *We feel a detailed discussion of this is outside the scope of this paper, which aims to describe the development of the tool kit. We are working on a second paper describing the initial experiences with its use and the raised concerns will be examined in more detail in this second paper.*

4-The authors mention the need to constantly update the toolkit to adapt to new research evidence. How should this be undertaken? Should the whole consultation process outlined in the manuscript be reproduced? It would be useful if the authors could provide more details as to how they envisage this.

*Indeed, as new evidence emerges the expert recommendations become potentially outdated. We have clearly identified this as a limitation of the toolkit in its current form. While we acknowledge the need to update the toolkit, we don’t want to provide any guidance on how to do this in terms of methodology as this feels outside the scope of this manuscript. We have however expanded on this theme in the limitation section for clarity. It readds as follows (line 410-413):*

*“Secondly, experts evaluated evidence available at the time, but research is a dynamically developing field. There is a need to iteratively update the OAT to reflect current evidence for best practice. How this can be done efficiently is subject to further work.”*

5- The limitation section could be expanded. Specifically, a critical assessment of the limitations of the consultation and Delphi approaches would need to be outlined.

*We have expanded on the limitations and have added as follows:*

*Third, the Delphi process was lengthy and demanding, which may have resulted in experts providing responses without the same level of careful consideration that they might have given in a shorter format*

Minor points
6- I seems there is a typo in a table2, line 3, middle column: the text is duplicated from the previous row.

*We have corrected this now.*

7- I did not understand the purpose of the approaches tool: I would invite the authors to reformulate the corresponding section for better clarity.

*The approaches tools aims to move from policy making to supporting implementation. Though those are two process we acknowledge that implementation considerations often influence the policy process directly. The approaches tool aims to bridge this by stage by combining planning for policy change and implementation.*

Reviewer #2:

1-It would interesting to understand why only 3 NMP contributed to this exercise? how representative are they really?

*Three NMPs were selected based on their expressed interest to participant in the tool development. The overall number of participating NMPs was also informed by trying to balance the need for representation and geographic variation and feasibility in terms of regular meetings and meaningful impact.*

2- Likewise it would interesting to know on what criteria were the so-called "experts" selected?

*We have outlined the criteria for the expert selection the method section. Experts had more than 10 years of professional experience in malaria research, a high-level expertise in malaria demonstrated by research publications on vivax malaria and/or health system strengthening, as well as capacity and willingness to contribute. Gender balance, and representation from different countries within the Asia Pacific region was considered.*

*Given the reviewer puts the term experts into quotation marks we assume they are worried about potential bias in the selection. We have added this to the limitations as suggested by reviewer #1. We also discuss the selection and compassion of expert when discussion different views of experts and NMP members on factors included in the OAT, which is likely based on their different background and understanding of contexts.*

3-The fact that some of the "options" such as the high-dose primaquine short regimen, were removed, despite the evidence that it is safe, it problematic.

*We agree that this was a problematic situation for the research team to navigate and highlights what reviewer #1 highlighted that evidence can be interpreted differently depending on many factors, some of them the appetite and willingness to embrace risk. For clarity we would like to highlight that the WHO recommendation against high dose short course PQ came out in November 2022 just one month before the OAT was presented to NMPs in December 2022. It was felt that providing a treatment option with a WHO recommendations against it would have put NMPs in a very difficult position. Expert were therefore approached again and* *asked to select the next best options if PQ7 (high dose) is not available. This option was then presented to NMP. In the submitted manuscript however the original selections are presented.*

4-Would be also very interesting to know the percentage of the NMP people who really understand P.vivax malaria. And what proportion actually have access to the scientific evidence?

*We are not entirely sure what the reviewer means with that and how one would define ‘a real understanding P. vivax malaria’ and how this impacts our findings. we assume that the reviewer might be concerned about how the level of knowledge on vivax malaria might have influenced their replies. We like to highlight that expert for the Delhi process were selected based a priory defined criteria and NMP co-developer were not among the selected experts. They were however instrumental as co-developers given their in-depth experience and knowledge of the policy making and implementation.*
